# Case Study: Intra-Patient Heterogeneity of Aneurysmal Tissue Properties

**DOI:** 10.3389/fcvm.2018.00082

**Published:** 2018-07-03

**Authors:** Giampaolo Martufi, Arianna Forneris, Samaneh Nobakht, Kristina D. Rinker, Randy D. Moore, Elena S. Di Martino

**Affiliations:** ^1^Department of Civil Engineering, University of Calgary, Calgary, AB, Canada; ^2^Unit for Health Innovation, School for Technology and Health, Royal Institute of Technology, KTH, Huddinge, Sweden; ^3^Biomedical Engineering Graduate Program, University of Calgary, Calgary, AB, Canada; ^4^Department of Chemical and Petroleum Engineering, University of Calgary, Calgary, AB, Canada; ^5^Centre for Bioengineering Research and Education and Libin Cardiovascular Institute of Alberta, University of Calgary, Calgary, AB, Canada; ^6^Department of Surgery, University of Calgary, Calgary, AB, Canada

**Keywords:** abdominal aortic aneurysm, histology, mechanical properties, FEA, CFD

## Abstract

**Introduction:** Current recommendations for surgical treatment of abdominal aortic aneurysms (AAAs) rely on the assessment of aortic diameter as a marker for risk of rupture. The use of aortic size alone may overlook the role that vessel heterogeneity plays in aneurysmal progression and rupture risk. The aim of the current study was to investigate intra-patient heterogeneity of mechanical and fluid mechanical stresses on the aortic wall and wall tissue histopathology from tissue collected at the time of surgical repair.

**Methods:** Finite element analysis (FEA) and computational fluid dynamics (CFD) simulations were used to predict the mechanical wall stress and the wall shear stress fields for a non-ruptured aneurysm 2 weeks prior to scheduled surgery. During open repair surgery one specimen partitioned into different regions was collected from the patient's diseased aorta according to a pre-operative map. Histological analysis and mechanical testing were performed on the aortic samples and the results were compared with the predicted stresses.

**Results:** The preoperative simulations highlighted the presence of altered local hemodynamics particularly at the proximal segment of the left anterior area of the aneurysm. Results from the post-operative assessment on the surgical samples revealed a considerable heterogeneity throughout the aortic wall. There was a positive correlation between elastin fragmentation and collagen content in the media. The tensile tests demonstrated a good prediction of the locally varying constitutive model properties predicted using geometrical variables, i.e., wall thickness and thrombus thickness.

**Conclusions:** The observed large regional differences highlight the local response of the tissue to both mechanical and biological factors. Aortic size alone appears to be insufficient to characterize the large degree of heterogeneity in the aneurysmal wall. Local assessment of wall vulnerability may provide better risk of rupture predictions.

## Introduction

An abdominal aortic aneurysm (AAA) is a localized dilatation of the abdominal aorta most often found in the infrarenal region of the artery above the iliac bifurcation. Both open and endovascular surgeries—the only treatments available—carry a significant risk of complications and should be reserved for cases that are at risk for rupture. The ideal diagnostic tool should reliably identify and rank the risk for rupture of an individual aorta, helping the design of clinical/surgical interventions and the selection of patients. Current clinical guidelines rely on the assessment of maximum aortic diameter as indication for surgical repair, overlooking the role played by wall heterogeneity and localized weakening (1).

Studies show that irreversible pathological remodeling of the extracellular matrix and structural degradation of the aortic wall trigger aortic dilatation, while inflammation and imbalance between elastin and collagen turnover are thought to be important biological processes involved in aneurysmal progression and rupture (2–4). There is growing evidence in the literature that abnormal blood flow patterns (5, 6) and high stresses (7) experienced by the diseased wall are important factors in the development of an aneurysm. Several investigations associated thrombus formation to disturbed hemodynamics, with regions of expansion and rupture characterized by low wall shear stress (WSS < 0.4 Pa) and intraluminal thrombus (ILT) accumulation (8, 9). Increased mechanical stress and ILT deposition, degradation of the elastic fibers and loss of integrity through inflammatory processes may lead to a reduction in the wall strength and, eventually, to rupture.

The present study aims at investigating intra-patient heterogeneity of mechanical and fluid mechanical stresses on the aortic wall and wall tissue histopathology on corresponding aneurysmal regions collected at the time of surgical repair. Specifically, the mechanical stress and the time-averaged wall shear stress (TAWSS) were predicted 2 weeks before surgical repair by employing finite element stress analysis (FEA) and computational fluid dynamics (CFD) simulations, respectively. The derived stress maps allowed for an informed collection of regional samples from the patient diseased aorta at the time of surgery. Finally, in the post-operative setting, histological analysis, and mechanical testing were performed on the harvested tissue and compared to the predicted fluid-mechanical stresses.

## Methods

One patient (age range 52–58 years) presenting with a non-ruptured infra-renal AAA with maximum diameter of 56.70 mm was included in the study after obtaining informed consent. The patient underwent routine contrast-enhanced computed tomography-angiography (CTA) examination 2 weeks prior to the scheduled surgery for aortic resection, following informed consent according to institutional ethical guidelines.

The three-dimensional AAA geometry was reconstructed from the stack of CTA images and pre-operative FEA and CFD simulations were performed to evaluate the patient-specific state of mechanical and fluid dynamic stresses. Tissue samples from different regions of the aneurysm were obtained fresh from the operating room according to an approved ethical protocol and used for post-operative assessment that included mechanical testing and histological analysis.

### Pre-operative simulations

#### CFD analysis

##### Computational model

The three-dimensional patient-specific aneurysmal lumen was reconstructed using an image-processing and model generation software (ScanIP; Simpleware Ltd., Exeter, UK). The mesh generating module (ScanFE; Simpleware Ltd., Exeter, UK) was used to discretize the reconstructed vessel geometry into a grid of tetrahedral elements with boundary layer to refine the mesh at the wall. The model was then imported in the commercial software Fluent (Ansys, Canonsburg, PA, USA) for CFD simulations. A sensitivity analysis was performed to assess the spatial resolution, in terms of mesh size, and temporal resolution, in terms of time step size. An isotropic, incompressible, Newtonian fluid was adopted to model the blood assuming a constant density of 1050 Kg/m^3^ and a constant dynamic viscosity of 0.00319 Pa·s.

A velocity inlet and two pressure outlets, corresponding to the iliac arteries, were imposed as boundary conditions. No-slip conditions were applied to the luminal surface. The pressure waveform applied at the outlets was computed by using a coupled 3-element Windkessel 0D model of the downstream vasculature.

The aortic wall was assumed to be rigid, although some of the features of wall elasticity were captured by coupling the Windkessel model to the 3D geometry.

##### Hemodynamic wall descriptors

Wall shear stress-based hemodynamic descriptors, namely the TAWSS, the oscillatory shear index (OSI) and relative residence time (RRT) were computed to characterize the blood flow patterns and quantify the hemodynamic disturbances. The TAWSS is the wall shear stress magnitude averaged over the cardiac cycle T:

$$TAWSS=\frac{1}{T}\int_{0}^{T}|WSS\left(s,t\right)|dt$$

*s* identifies the position on the vessel wall at time instant *t*.

The OSI provides a measurement of the WSS vector deviation from the main direction of the flow over the cardiac cycle:

$$OSI=0.5\;\left[1-\left(\frac{|\int_{0}^{T}WSS\left(s,\;t\right)dt|}{\int_{0}^{T}|WSS\left(s,\;t\right)|dt}\right)\right]$$

Finally, the RRT is defined as a combination of the previous parameters and provides an information about the time spent by the blood particles near the vessel wall:

$$RRT=\frac{1}{\left(1-2\;\cdot \;OSI\right)\;\cdot \;TAWSS}$$

#### Stress analysis

##### Constitutive model of aneurysmal wall

The aneurysmal wall was modeled as a fibrous collagenous tissue, where bundles of collagen fibrils mutually cross-linked by proteoglycans (CFPG-complex) reinforce an isotropic matrix material (elastin and ground matrix). The matrix material was described by an isotropic Neo-Hookean constitutive model. The CFPG-complex was described by a virtually linear stress-strain response and a triangular probability density function that defines the relative amount of engaged collagen fibrils when the collagen fiber is exposed to stretch λ. An isotropic constitutive model was used for the fibril and a constant collagen fiber density $\frac{{\rho \left(\text{N}\right)=\rho }_{0}=\;1.35}{4\pi }{\;sr}^{-1}$ was adopted in all directions. The model integrates two mechanical parameters (μ and *k)*, and one structural parameter (λ_max_). In details, the mechanical parameters μ and *k* quantify the matrix material shear modulus and the stiffness of the CFPG-complex, respectively, while the value of λ_max_ characterizes the degree of waviness of the collagen fibrils. The detailed model and its numerical implementation are reported in Martufi and Gasser (10).

##### Geometry, load, and boundary conditions

The aortic geometry was reconstructed from the CTA data (A4research, VASCOPS GmbH) acquired prior to elective repair.

A suite of custom-written routines (MATLAB 2014a, The MathWorks, Inc., Natick, Massachusetts, USA) was used to segment the lumen, the outer and inner wall of the vessel. The minimum distances between the inner and outer wall and between the inner wall and the lumen were used to estimate the AAA wall thickness and the ILT thickness, respectively (11, 12). These estimates were used to redefine the hexahedral elements that discretize the aortic wall segmented in VASCOPS in order to account for the local wall thickness (13).

The top and bottom surfaces of the Finite Element model were fixed, no contact with the surrounding organs was considered and peak systolic blood pressure of 120 mmHg (16 kPa) was applied.

The maximum principal stress (MPS) field was predicted by means of finite element analysis (FEA) using FEAP (vs. 8.2, University of California at Berkeley, CA, USA) considering non-homogeneous population-averaged material properties (13) and using the described constitutive model. In details, each element was considered as belonging to one of four categories with their constitutive parameters assigned according to a discrete categorization of the material properties, i.e., thin wall-thin ILT (μ = 50 kPa, *k* = 7,800 kPa, λ_max_ = 1.04), thin wall-thick ILT (μ = 5 kPa, *k* = 3,600 kPa, λ_max_ = 1.04), thick wall-thin ILT (μ = 40 kPa, *k* = 7,400 kPa, λ_max_ = 1.12), and thick wall-thick ILT (μ = 5 kPa, *k* = 3,900 kPa, λ_max_ = 1.12) (13). Finally, ILT tissue was modeled using a one parameter Ogden-like strain energy function, with 2.11 kPa as constitutive parameter (14).

### Intra-operative tissue collection

#### Surgery map definition

Twenty-five different regions were identified on the aneurysmal sac, i.e., 12 left lateral regions (six left anterior and six left posterior), 12 right lateral regions (six anterior and six posterior) and the neck region. Region-averaged maximum principal stress (*MPS*_*ra*_), region-averaged time averaged wall shear stress (*TAWSS*_*ra*_), region-averaged oscillatory shear index (*OSI*_*ra*_), region-averaged relative residence time (*RRT*_*ra*_), region-averaged ILT (*ILT*_*ra*_), and wall thickness (*WT*_*ra*_) were computed and used as global descriptors of the geometrical and mechanical characteristics for each region.

**Figure 1:**
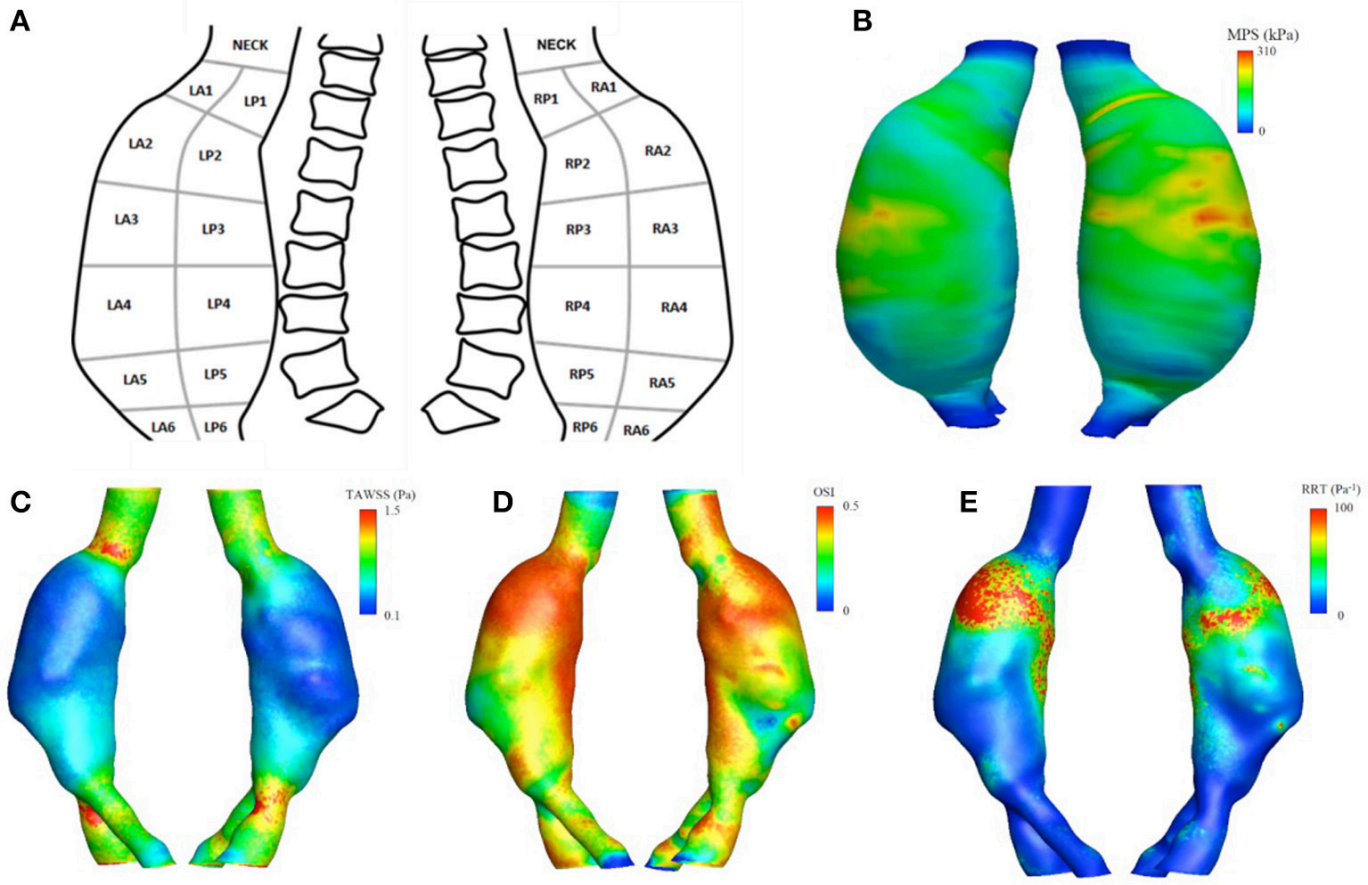
Upper panel **(A)** schematic representation of the surgery map used for tissue collection. **(B)** The maximum principal stress (MPS) field obtained from the finite element analysis. Lower panel: aortic luminal surface distribution of WSS-based hemodynamic descriptors, in detail **(C)** TAWSS, **(D)** OSI, **(E)** RRT.

The defined regions served as a guide to collect tissue from the patient resected aorta corresponding to areas with different global descriptors (Figure 1A and Table 1).

**Table 1:** Summary of geometrical and mechanical parameters of the different aneurysmal regions.

**Region ID**	**WT_ra_ (mm)**	**ILT_ra_ (mm)**	**TAWSS_ra_ (Pa)**	**OSI_ra_**	**RRT_ra_(Pa^−1^)**	**MPS_ra_ (kPa)**
NECK	1.3 ± 0.1	0	0.60	0.25	5.1	36.0
RP1	1.5 ± 0.2	4.5 ± 1.0	0.45	0.44	25.4	120.5
RA1	1.4 ± 0.1	3.1 ± 1.4	0.49	0.34	6.0	117.4
RP2	1.3 ± 0.1	3.8 ± 0.5	0.41	0.40	14.4	149.2
RA2	1.3 ± 0.1	5.5 ± 1.3	0.32	0.45	54.7	153.6
RP3	1.4 ± 0.1	6.3 ± 1.8	0.16	0.46	59.6	145.7
RA3	1.4 ± 0.1	0	0.18	0.40	36.7	202.9
RP4	1.4 ± 0.1	10.4 ± 1.0	0.13	0.44	47.1	115.0
RA4	1.7 ± 0.2	7.0 ± 2.0	0.09	0.31	16.4	138.4
RP5	1.4 ± 0.1	10.0 ± 1.3	0.19	0.43	24.8	79.4
RA5	1.3 ± 0.1	8.7 ± 1.1	0.15	0.22	8.4	91.1
RP6	1.4 ± 0.1	6.5 ± 0.7	0.40	0.35	5.4	87.2
RA6	1.2 ± 0.1	6.5 ± 0.9	0.31	0.29	4.9	86.6
LA1	1.5 ± 0.2	6.4 ± 1.4	0.41	0.43	38.5	101.6
LP1	1.4 ± 0.1	4.5 ± 0.5	0.55	0.24	2.3	85.7
LA2	1.4 ± 0.1	7.3 ± 0.9	0.17	0.47	109.8	100.4
LP2	1.3 ± 0.1	7.0 ± 1.2	0.43	0.46	45.9	143.6
LA3	1.4 ± 0.1	4.6 ± 1.8	0.15	0.41	42.4	166.7
LP3	1.4 ± 0.1	8.6 ± 1.1	0.18	0.46	67.9	89.9
LA4	1.6 ± 0.1	4.7 ± 2.6	0.12	0.31	12.0	158.4
LP4	1.5 ± 0.2	10.2 ± 0.7	0.16	0.46	64.1	82.0
LA5	1.4 ± 0.1	8.8 ± 1.8	0.18	0.29	7.1	91.7
LP5	1.4 ± 0.1	10.8 ± 0.9	0.19	0.42	19.8	76.1
LA6	1.5 ± 0.1	8.0 ± 1.0	0.23	0.35	10.9	74.2
LP6	1.5 ± 0.1	7.9 ± 0.6	0.38	0.36	5.8	74.6

*Region-averaged intraluminal thrombus (ILT_ra_); region-averaged wall thickness (WT_ra_) estimated from images; region-averaged time average wall shear stress (TAWSS_ra_); region-averaged oscillatory shear index (OSI_ra_); region-averaged relative residence time (RRT_ra_); region-averaged maximum principal stress (MPS_ra_)*.

#### Tissue collection

From the intra-operative tissue specimen, ten regions (~20 × 15 mm) were obtained; each was ~20 × 15 mm in size with one edge parallel to the circumferential orientation with respect to the intact aorta and the other one perpendicular to it. Tissues that were selected for histology were fixed in 10% buffered formalin immediately after collection. Tissues destined for mechanical analysis were placed in saline solution and refrigerated at 4°C.

### Post-operative assessment

#### Mechanical tensile tests

Four regions ~10 × 10 mm in size were tested within 48 h of procurement: one from the neck, two from the right anterior region and one from the left anterior region. Width, length, and thickness were measured for each sample with a manual caliper. Tissues were tested using a planar biaxial system (ElectroForce, TA Instruments, MO, USA) with displacement-controlled protocol reaching a maximum stretch of 40% on both axes while being continuously irrigated with saline solution at 37°C. After a short thermal equilibration period, a frequency of 0.333 Hz and 10 cycles count were used for each experiment. If the tissue sample was not wide enough to perform biaxial tensile tests, uniaxial tensile test at the same strain rate and with the same maximum stretch was performed. From the acquired data, the first Piola-Kirchhoff stress was calculated normalizing the recorded force by the cross-sectional area in the undeformed configuration, while the stretch was computed as the deformed length normalized by the original specimen length.

#### Constitutive model for the individual specimen properties

A computational model representing a single cubic element of tissue was used to estimate the mechanical parameters μ*, k*, and the structural parameter λ_max_ of the constitutive model from the tensile experimental data. The clear physical meaning of the model parameters allowed their straightforward identification by manual adjustments as described previously (10). For each specimen, the stress values obtained in the physiological strain range for an aneurysm [~7%, see Satriano et al. (15)] using the optimized *ex-vivo* material parameters were compared with stresses obtained using our previously published population-averaged material properties (7). The root-mean-square error (RMSE) was employed to measure differences.

#### Histological analysis

Samples for histology were fixed in 10% buffered formalin and then stained with Movat and Picrosirius red to assess elastin fragmentation and quantify the percentage of total area occupied by collagen fibers. Slides were observed and imaged at 2X and 20X magnification using an Olympus BX53 DP73 microscope.

Visual inspection was used to identify the tunica media from Movat images at 2X magnification. The pixels identified as belonging to the media were then used to estimate the percentage of area occupied by collagen in the corresponding Picrosirius red-stained slides (16). The original images were first converted into grayscale images, i.e., the luminance (luminous intensity per unit area) of the original image was retained while information about hue and saturation were eliminated. In order to highlight the collagen structure, brightness and contrast were automatically optimized according to the histograms and the images were finally converted into binary images. The percentage area of collagen in the media was computed as the percentage of pixels identified as collagen material.

Elastin fragmentation in the media was visually inspected from Movat stained images at 20X magnification and scored as (1) minor, (2) moderate, or (3) high. A score of (4) was assigned if no elastin was detected in the sample and N/A if the media was entirely disrupted.

## Results

### Pre-operative simulations

Figure 1 (lower panels) show the luminal distribution of WSS-based hemodynamic descriptors allowing for a visual inspection of flow patterns within the vessel. The aneurysmal region presented lower values of TAWSS (Figure 1C), compared to those observed in the areas upstream and downstream the dilatation, between 0.1 and 0.4 vs. 0.8 and 1.5 Pa.

The values for the OSI (Figure 1D) ranged between 0 and 0.5, while the RRT (Figure 1E) varied between 10 and 100 Pa^−1^ within the aneurysmal region, with higher values located in the proximal segment of the left lateral region.

The MPS field, computed accounting for locally varying wall thickness and non-homogenous material properties, is plotted in Figure 1B. The predicted macroscopic stress was distributed non-uniformly over the aneurysmal wall. High stresses were located at ILT-free regions with thin wall and regions with thick wall covered with thin ILT.

There was a statistically significant negative correlation between region-averaged maximum principal stress (*MPS*_*ra*_) and *ILT*_*ra*_ (Pearson's ρ = −0.51, *p* = 0.009), between *MPS*_*ra*_ and *TAWSS*_*ra*_ (Pearson's ρ = −0.55, *p* = 0.005), and between *MPS*_*ra*_ and *RRT*_*ra*_ (Pearson's ρ = −0.41, *p* = 0.04). The maximum *MPS*_*ra*_ of 202.9 kPa was recorded in the RA3 region, where an ILT-free wall 1.4 ± 0.1 mm thick was measured. In contrast, the lowest *TAWSS*_*ra*_ (0.09 Pa) was observed in region RA4, where a thick wall of 1.7 ± 0.2 mm covered by 7.0 ± 2.0 mm of *ILT*_*ra*_ was recorded. The maximum *OSI*_*ra*_ and *RRT*_*ra*_ were 0.47 and 109.8 Pa^−1^, respectively, and were both localized in the LA2 region (*WT*_*ra*_ = 1.4 ± 0.1 mm and *ILT*_*ra*_ = 7.3 ± 0.9 mm). Finally, the maximum *TAWSS*_*ra*_ (0.60 Pa) and minimum *MPS*_*ra*_ (36.0 kPa) were located in the neck region (see Table 1).

### Post-operative assessment

#### Mechanical properties

Tissue samples harvested from the neck and right anterior region RA4 were subject to uniaxial tensile testing. Samples collected from regions RA2 and LA4 underwent biaxial tensile tests. Following the categorization based on wall and ILT thickness (13), sample from RA4 was labeled as tissue with thick wall and thick ILT (*WT*_*ra*_ > 1.4 mm, *ILT*_*ra*_ > 6.5 mm), RA2 belonged to the category with thin wall and thin ILT and LA4 was classified as thick wall, thin ILT. Because the neck region was ILT-free no category was assigned.

**Figure 2:**
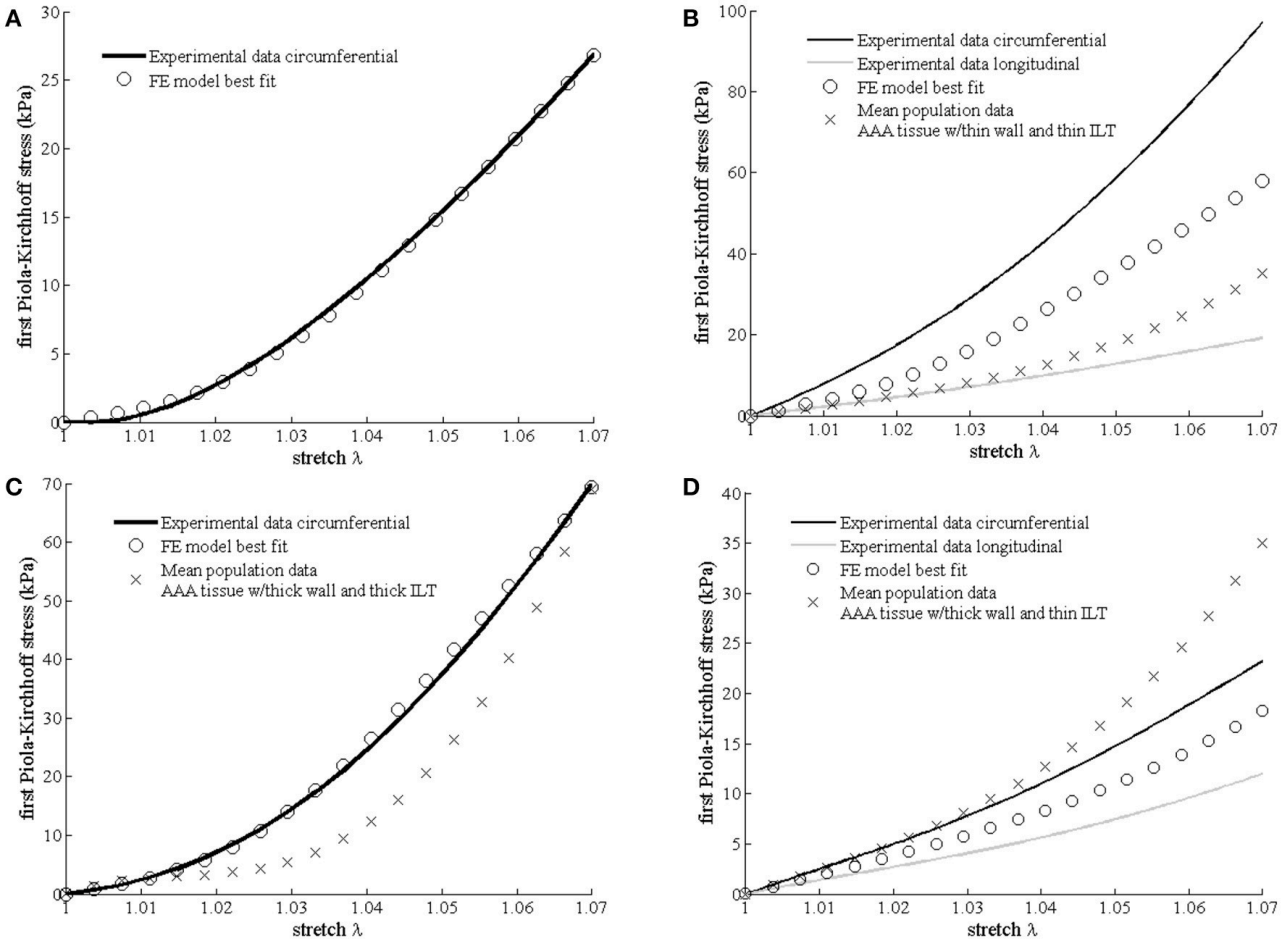
Differences in mechanical properties between samples harvested from different aneurysmal regions. Experimental data, best fit Finite Element (FE) results (open circles) according to the multi-scale constitutive model and mean population data (cross) derived from Martufi et al. (13) are shown for each region. **(A)** Neck region (no mean population data available); **(B)** right-anterior region RA2; **(C)** right-anterior region RA4; and **(D)** left-anterior region LA4.

Figure 2 shows the experimental mechanical response, the FE model best fit and the stress-stretch predictions using a population based mean material properties according to the above defined subgroups (13).

The fitted values for the matrix shear modulus (μ) of the samples collected from the right-anterior region, were 60 kPa for RA2 and 10 kPa for RA4, with a CFPG-complex stiffness (*k*) of 3,500 and 1,000 kPa, respectively. The estimated μ and k parameters of the constitutive model were 32 and 2,900 kPa, respectively for the specimen harvested from the left-anterior region LA4, and 8 and 2,500 kPa for the neck tissue sample. Table 2 reports a summary of the mechanical parameters found. Neighboring regions are reported to facilitate comparison with histological results. It must be noted that comparisons between neighboring regions are indicative due to the high variability. Specimen RA2 showed the highest CFPG-complex stiffness and is adjacent to RA1 that shows high collagen content. The neck specimen allowed a direct comparison and showed the lowest elastin fragmentation, the lowest matrix material shear modulus and the highest λ_max_ suggesting high elastin content.

**Table 2:** Summary of structural and mechanical parameters of the constitutive model for different aneurysmal regions.

**Region ID**	**Matrix material shear modulus μ (kPa)**	**CFPG-complex stiffness *k* (kPa)**	**λ_max_**	**Adjacent regions (refer to Table 3 for histology)**
NECK	8	2,500	1.27	NECK
RA2	60	3,500	1.04	RA1
RA4	10	1,000	1.06	RP4, RA5
LA4	32	2,900	1.13	LA5, LA3

*Adjacent regions are reported to facilitate comparisons with histological results in Table 3*.

#### Pre-operative mechanical properties assumptions and specimen individual tissue properties

For the three samples tested, a total RMSE of 8.8 kPa was recorded between the actual stress values and those estimated in the preoperative setting using population-averaged material properties. RA2 exhibited the largest RMSE (13.7 kPa). The RMSE decreased to the values of 6.8 and 0.95 kPa, for LA4 and RA4, respectively. No comparison was performed for neck tissue because population-averaged parameters were not available.

#### Histology

**Figure 3:**
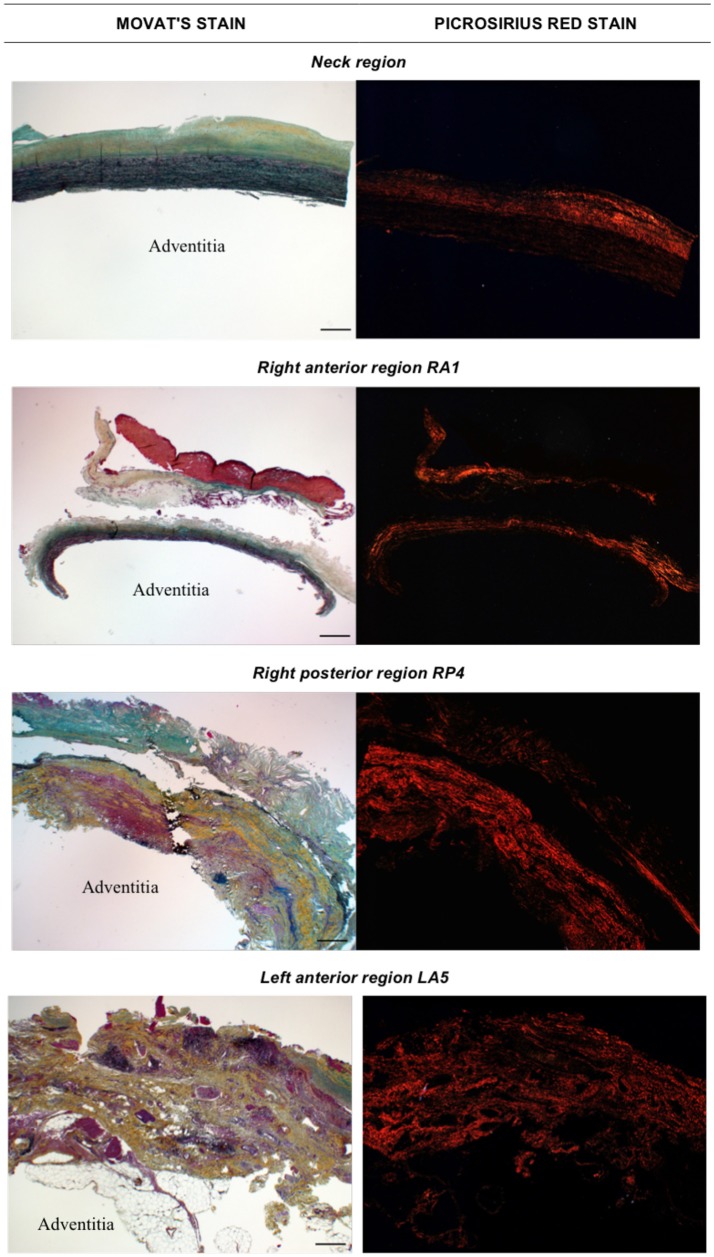
Histological images obtained with Movat stain (left) and Picrosirius red stain (right) at 2X magnification. The adventitia layer of the aortic wall is labeled as reference in each image. Scale bar is 0.5 mm (located bottom right).

Movat and Picrosirius red stained samples at 2X magnification are showed in Figure 3.

**Figure 4:**
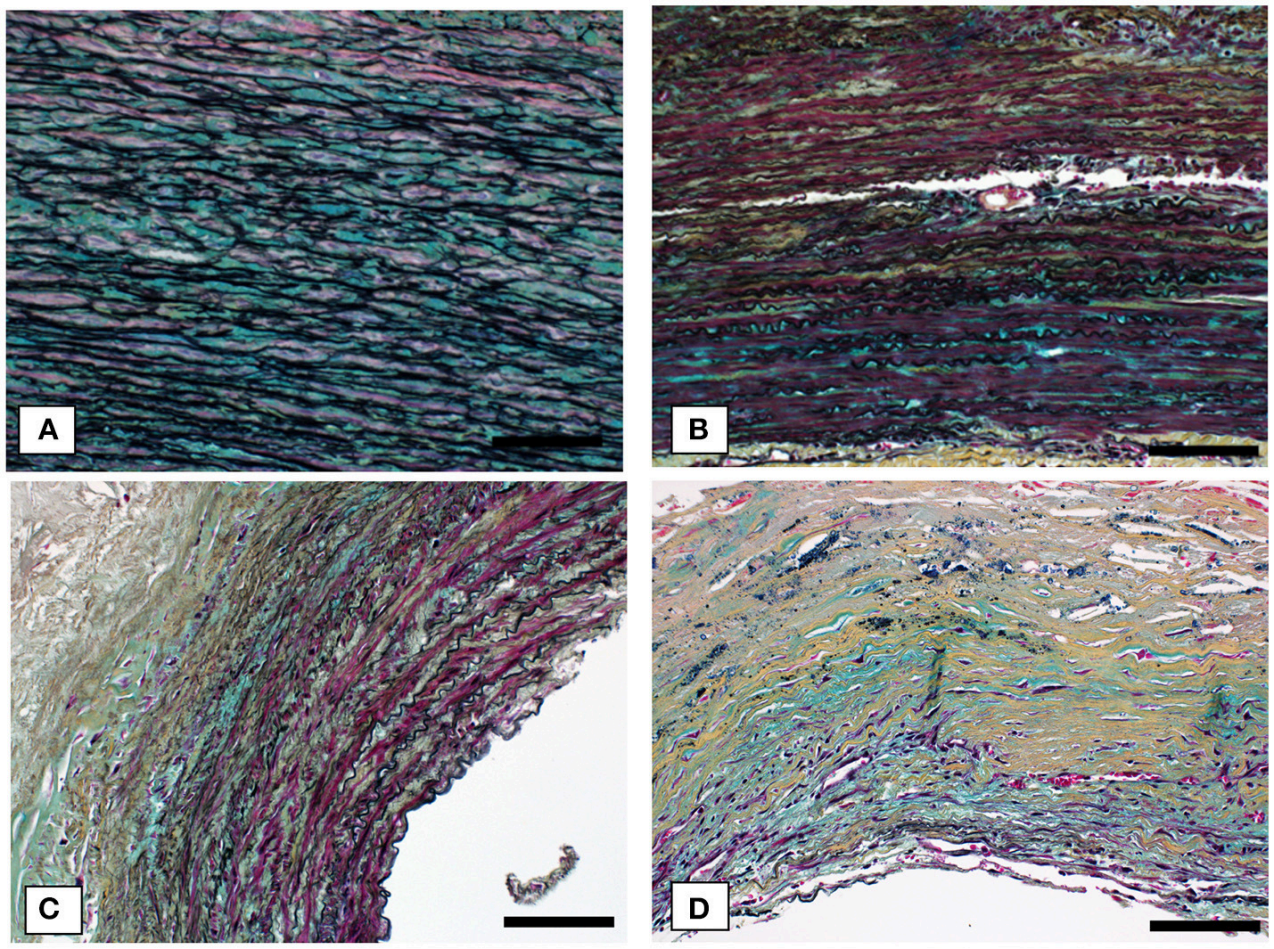
Representative cases of elastin fragmentation in the media. Movat images at 20X magnification: **(A)** Neck region with low elastin fragmentation (score 1); **(B)** LA3 region with moderate elastin fragmentation (score 2); **(C)** RA1 region with high elastin fragmentation (score 3) and **(D)** RA5 region with no elastin content (score 4). Scale bar 0.1 mm.

**Figure 5:**
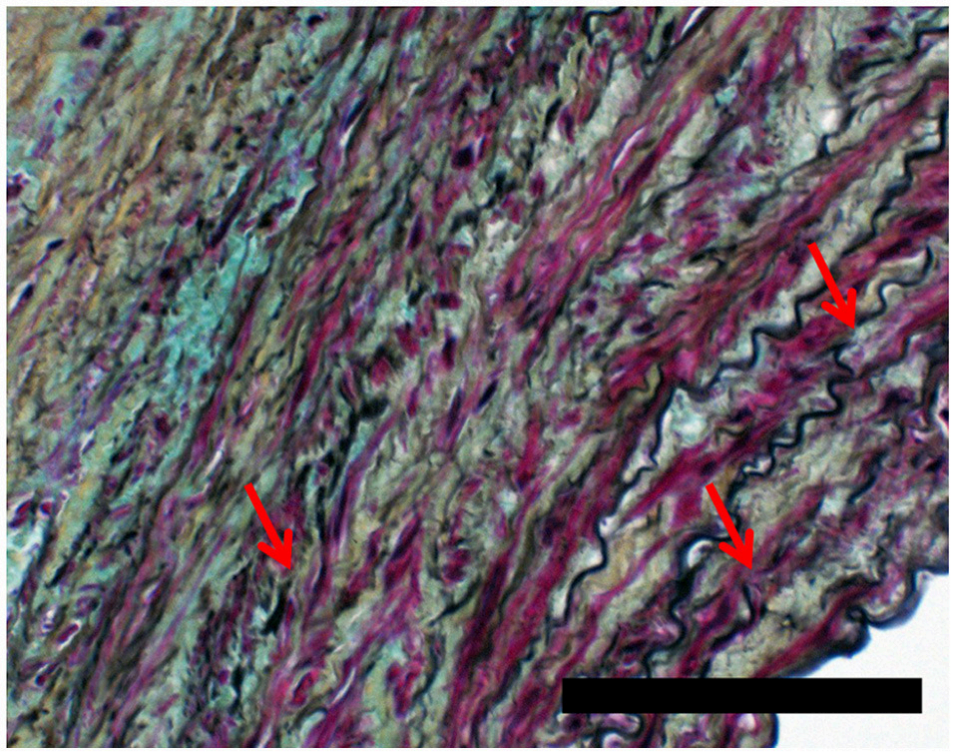
Detail of RA1 sample (20x) showing elastin fragmentation (red arrows). Scale bar 0.1 mm.

Figures 4, 5 show Movat's stain at 20X magnification for four regions to demonstrate the degree of elastin fragmentation in the media. There was a strong positive correlation between elastin fragmentation score and percentage of collagen content in the media (Pearson's ρ = 0.94, *p* = 0.019). RP4 and LA5 resulted in score 4 for elastin fragmentation due to the complete disruption of the medial layer. The two highest collagen contents were measured in RA5 (65.8%) and RA1 (38.2%) where a score of 4 and 3 for elastin fragmentation was recorded, respectively. Both LA1 and LA3 exhibited moderate elastin disruption (score 2); however, collagen content was higher for LA1 (19.7%) than LA3 (10.8%). Finally, low elastin fragmentation (score 1) with collagen content of 12.4% was observed for the neck sample (see Table 3 and Figures 3, 4).

**Table 3:** Summary of geometrical, mechanical and histological features for different aneurysmal regions.

**Region ID**	**WT_ra_ (mm)**	**ILT_ra_ (mm)**	**TAWSS_ra_ (Pa)**	**OSI_ra_**	**RRT_ra_ (Pa^−1^)**	**MPS_ra_ (kPa)**	**Collagen content (%)**	**Elastin fragmentation**
NECK	1.3	0	0.60	0.25	5.06	36.05	12.4	1
RA1	1.4 ± 0.1	3.1 ± 1.4	0.49	0.34	6.04	117.4	38.2	3
RP4	1.4 ± 0.1	10.4 ± 1.0	0.13	0.44	47.14	115.0	N/A	N/A
RA5	1.3 ± 0.1	8.7 ± 1.1	0.15	0.22	8.42	91.1	65.8	4 (no elastin)
LA1	1.5 ± 0.2	6.4 ± 1.4	0.41	0.43	38.46	101.6	19.7	2
LA3	1.4 ± 0.1	4.6 ± 1.8	0.15	0.41	42.44	166.7	10.8	2
LA5	1.4 ± 0.1	8.8 ± 1.8	0.18	0.29	7.11	91.6	N/A	N/A

### Comparisons

Statistically non-significant negative correlations were found between *TAWSS*_*ra*_ and elastin fragmentation (Pearson's ρ = −0.62, *p* = 0.18). A complete media disruption (N/A for elastin fragmentation) was observed for RP4 where low *TAWSS*_*ra*_ (0.13 Pa), high *OSI*_*ra*_ (0.44), and *RRT*_*ra*_ (47.14 Pa^−1^) and thick ILT (10.4 ± 1.0 mm) were measured. A *TAWSS*_*ra*_ of 0.15 Pa was associated with both moderate elastin fragmentation (score 2) and entirely degraded elastin (score 4) in LA3 and RA5, respectively.

Despite exhibiting the same *TAWSS*_*ra*_, RA5 presented an *OSI*_*ra*_ almost half than the one measured for LA3 (0.22 vs. 0.41) and five times smaller *RRT*_*ra*_ (8.42 vs. 42.44 Pa^−1^). Additionally, RA5 showed an ILT deposition almost double than the one measured for LA3, i.e., *ILT*_*ra*_ = 8.7 ± 1.1 mm for RA5 vs. *ILT*_*ra*_ = 4.6 ± 1.8 mm for LA3, and six times higher collagen content, i.e., 65.8% for RA5 vs. 10.8% for LA3. LA5 presented a *ILT*_*ra*_ of 8.8 ± 1.8 mm similar to RA5 that was associated with a *TAWSS*_*ra*_ of 0.18 Pa (*OSI*_*ra*_ = 0.29;*RRT*_*ra*_ = 7.11 Pa^−1^), and a complete disruption of the media layer (N/A for elastin fragmentation). LA1 showed a moderate elastin fragmentation (score 2) with *TAWSS*_*ra*_ of 0.41 Pa, *OSI*_*ra*_ of 0.43 and *RRT*_*ra*_ of 38.46 Pa while, for RA1 the *TAWSS*_*ra*_ of 0.49 Pa (*OSI*_*ra*_ = 0.34; *RRT*_*ra*_ = 6.04 Pa) was related to higher elastin fragmentation (score 3). Finally, the neck region displayed the lowest elastin fragmentation score (1) with the lowest *RRT*_*ra*_ (Pa^−1^) and the highest *TAWSS*_*ra*_ (0.60 Pa).

There was no correlation between *MPS*_*ra*_ and collagen content (Pearson's ρ = −0.08, *p* = 0.9). The neck region exhibited the lowest values of *MPS*_*ra*_ (36.0 ± 35.2 kPa) and the lowest score for elastin fragmentation (score 1) with relatively low percentage of collagen in the media (12.4%). RA5 and LA5 showed similar stress level (RA5: *MPS*_*ra*_ = 91.1 kPa; LA5: *MPS*_*ra*_ = 91.6 kPa) and similar ILT thickness deposited (RA5: *ILT*_*ra*_ = 8.7 ± 1.1 mm; LA5: *ILT*_*ra*_ = 8.8 ± 1.8 mm) that were associated with high elastin fragmentation or complete media destruction in both the samples, and with the highest collagen content (65.8%) for RA5. RA1 and RP4 were exposed to similar stress level (RA1: *MPS*_*ra*_ = 117.40 kPa; RP4: *MPS*_*ra*_ = 114.99 kPa) but presented different ILT coverage (RA1: *ILT*_*ra*_ = 3.1 ± 1.4 mm; RP4: *ILT*_*ra*_ = 10.4 ± 1.0 mm) that was associated with a score of 3 for elastin fragmentation and a collagen content of 38.2% for RA1, while RP4 had complete media destruction.

LA1 and LA3 showed a moderate fragmentation of elastic fibers (score 2) that was associated with different stress levels and different ILT thickness; i.e., LA1: *ILT*_*ra*_ = 6.4 ± 1.4 mm and *MPS*_*ra*_ = 101.64 kPa; LA3: *ILT*_*ra*_ = 4.6 ± 1.8 mm and *MPS*_*ra*_ = 166.67 kPa (see Table 3 and **Figures 3, 4**).

## Discussion and conclusion

An aortic aneurysm is the final result of a multifactorial process that involves pathological remodeling and degradation of the aortic wall leading to its weakening.

Early publications have reported potential correlation between mechanical stress and metabolism in the aneurysmal wall supporting the hypothesis of a stress mediated process that weakens the vessel (7, 17). However, evidences on the correlation between mechanical environment and underlying histological structure are currently very limited. This study focused on intra-patient variability of local mechanical and fluid dynamic stresses and on histological and mechanical properties of corresponding aneurysmal regions evaluated from one *ex-vivo* specimen.

The preoperative simulations provided insight on the stresses acting on the wall. Both MPS and WSS-based hemodynamic descriptors were distributed non-uniformly over the aneurysmal wall. The entire aneurysmal lumen generally presented disturbed flow conditions characterized by low oscillatory wall shear stress and high relative residence time pointing to recirculation and poor wash out associated with adverse remodeling of the extracellular matrix and ILT deposition. The effect of altered local hemodynamics was particularly evident in the proximal segment of the left anterior region, where LA2 and LA3 showed high *OSI*_*ra*_, high *RRT*_*ra*_, and low *TAWSS*_*ra*_ and were characterized by thick ILT.

The tensile tests on tissues collected demonstrated that constitutive parameters estimated non-invasively from geometric variables—ILT thickness and wall thickness, according to Martufi et al. (13)—produce a fairly accurate prediction of the *ex-vivo* constitutive model obtained fitting the experimental tensile tests data directly, suggesting a relationship between geometric macroscopic parameters and tissue properties.

The seven histological samples collected cover distal, central, and proximal regions and showed very heterogeneous features in terms of elastin degradation and collagen content. Higher degrees of medial disruption were found in the distal regions of the aneurysmal expansion.

Findings from the histological analysis showed a positive correlation between elastin fragmentation and collagen content in the media, suggesting a compensatory process that involves progressive increase in collagen synthesis and wall remodeling. It is hypothesized that as the aneurysm grows, the elastic fibers are distributed over a bigger area and elastin synthesis further decreases due to inflammatory processes and muscle cells apoptosis (18, 19). Elastin fragmentation was generally associated with low *TAWSS*_*ra*_and high *MPS*_*ra*_, but no statistically significant correlation was found.

The first limitation is that the analysis encompasses one aneurysmal specimen from one patient. This study was designed as a feasibility study to investigate the presence of significant heterogeneity in the aneurysm wall, even intra-patient.

The second limitation, pertaining the pre-operative stress analysis, is the use of population average parameters for the constitutive equations of both wall and ILT. The constitutive parameters were modulated locally based on Martufi et al. (13) and reasonable agreement was found between pre-operative and *ex-vivo* parameters as confirmed by the experimental tensile tests on explanted tissues.

Third, ILT was modeled as a non-porous material overlooking the complex role of the intraluminal thrombus as a biomechanically active component (20).

The constitutive model of the wall also presents limiting assumptions: the isotropic matrix model does not account directly for elastin degradation and smooth muscle cells (SMCs) are not directly modeled. However, the relative contribution of the isotropic component is reduced in the presence of elastin degradation. It should also be noted that SMCs are almost absent in the diseased aneurysmal wall at late stages (21).

Finally, the velocity boundary conditions at the inlet of the fluid dynamic model were not patient-specific and the aortic wall was assumed to be rigid during the simulation. Despite a typically stiffer wall, a compliant effect is still observable in aneurysmal patients; accordingly, some of the features of a compliant wall were captured by coupling the 3-element Windkessel model of the downstream vasculature.

Our findings point to the importance of the local mechanical environment in promoting intra-patient wall heterogeneity that is observable in the analysis of adjacent regions, such as RA3 and RA4. RA3 is an ILT-free region exhibiting the maximum *MPS*_*ra*_, while RA4, labeled as tissue with thick wall and thick ILT, presents the lowest *TAWSS*_*ra*_. The heterogeneity of the wall is confirmed by the postoperative assessment carried out by mean of mechanical tensile tests and histological analysis on the surgical specimen.

It is interesting to note that altered hemodynamics, and low TAWSS values appeared to be co-localized with wall exhibiting disrupted elastin and generally thicker ILT. While the low TAWSS values were computed at the interface between lumen and ILT, and not at the aneurysmal wall surface, it appears that altered fluid dynamics at the luminal surface co-localizes with disrupted wall constituents even in the presence of a thrombus layer, pointing to a remodeling mechanism that may be mediated by matrix proteinases secreted in the thrombus. This may help explain why altered hemodynamics and low TAWSS have been reported at sites of aneurysms rupture regardless of the presence of intraluminal thrombus (9).

The large variability observed at the local level in terms of mechanical properties and disruption of wall constituents suggests that local, rather than global, variables should be considered in the future for better risk of rupture prediction. Each individual aneurysmal region may have very different susceptibility to rupture depending on the combination of wall vulnerability and local fluid-mechanics conditions. True patient- and location-specific indicators of wall vulnerability are needed to increase the reliability of clinical outcomes prediction and consequent risk stratification as a mean for selective surgical repair.

## Ethics statement

This study was carried out in accordance with the recommendations of the Conjoint Health Research Ethics Board at the University of Calgary. All subjects gave written informed consent in accordance with the Declaration of Helsinki. The protocol was approved by the Conjoint Health Research Ethics Board.

## Author contributions

GM: FEA, tensile test analysis, histology, results comparison, manuscript preparation; AF: CFD simulation, CFD indices, and results, results comparison and discussion, manuscript preparation; SN: Windkessel model for CFD, CFD simulation; KR: conception and design of the study; RM: study design, patient selection, specimen collection; ED: conception and design of the study, critical evaluation of results, histology analysis, manuscript preparation. All authors contributed to manuscript revision, read, and approved the submitted version.

### Conflict of interest statement

The authors declare that the research was conducted in the absence of any commercial or financial relationships that could be construed as a potential conflict of interest.
